# Inhibition of IAPP Aggregation and Toxicity by Natural Products and Derivatives

**DOI:** 10.1155/2016/2046327

**Published:** 2015-11-15

**Authors:** Amit Pithadia, Jeffrey R. Brender, Carol A. Fierke, Ayyalusamy Ramamoorthy

**Affiliations:** Biophysics and Department of Chemistry, University of Michigan, Ann Arbor, MI 48109-1055, USA

## Abstract

Fibrillar aggregates of human islet amyloid polypeptide, hIAPP, a pathological feature seen in some diabetes patients, are a likely causative agent for pancreatic beta-cell toxicity, leading to a transition from a state of insulin resistance to type II diabetes through the loss of insulin producing beta-cells by hIAPP induced toxicity. Because of the probable link between hIAPP and the development of type II diabetes, there has been strong interest in developing reagents to study the aggregation of hIAPP and possible therapeutics to block its toxic effects. Natural products are a class of compounds with interesting pharmacological properties against amyloids which have made them interesting targets to study hIAPP. Specifically, the ability of polyphenolic natural products, EGCG, curcumin, and resveratrol, to modulate the aggregation of hIAPP is discussed. Furthermore, we have outlined possible mechanistic discoveries of the interaction of these small molecules with the peptide and how they may mitigate toxicity associated with peptide aggregation. These abundantly found agents have been long used to combat diseases for many years and may serve as useful templates toward developing therapeutics against hIAPP aggregation and toxicity.

## 1. Introduction

Human islet amyloid polypeptide (hIAPP) or amylin is a 37-residue peptide hormone secreted from *β*-cells within the islet of Langerhans in the pancreas. Physiologically, IAPP has a role in glucose metabolism by inhibiting insulin stimulated glucose uptake and glycogen synthesis as well as possible roles in gastric emptying and modulating insulin secretion [1, 2]. Beyond this normal physiological role, IAPP has received much attention due to its possible involvement in the pathology of diabetes mellitus, or type II diabetes [1, 2]. Comparisons of the pancreas of diabetic and nondiabetic individuals at the beginning of the 20th century revealed many (but not all) diabetics had large masses of insoluble protein of an unidentified protein that could be stained with iodine [3]. Later sequencing in the 1980s identified this protein in these deposits as the new hormone IAPP and further confirmed the deposits as amyloid fibers [4], a particular form of misfolded proteins which adopt a cross-*β* sheet structure with each monomer in the fiber adopting a *β* sheet structure and the *β* sheets of each monomer linked together by strong hydrogen bonds to create a long fiber.

**Figure 1:**
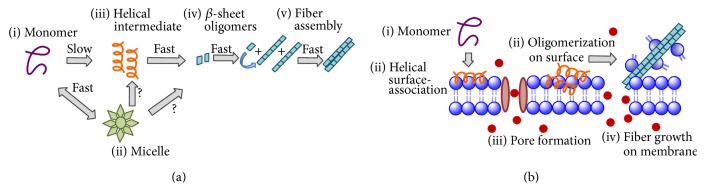
Simplified representation of hIAPP aggregation in solution (a) and on the membrane (b). In solution (a), hIAPP initially exists in most experiments as a monomeric peptide (i) in exchange with a micelle-like form (ii). Transient helical (iii) and *β*-sheet (iv) intermediates have been proposed but the exact nature and order of these species in the aggregation process are not clear. Once the nucleus for aggregation is formed, the final steps of the aggregation process are the elongation of the fiber by the addition of monomers (or possibly *β*-sheet oligomers) to the ends of the fiber and the lateral association of individual fiber strands (protofilaments) to form the amyloid fiber (v). On the membrane (b), monomeric hIAPP (i) can bind the membrane (ii) and self-associate on the membrane to form pores (iii) or helical structures (iv) that eventually form membrane-associated amyloid fibers (iv). During the fiber formation process (iv), lipids can be incorporated into the hydrophobic ends of the fiber which would be otherwise exposed to water, causing disruption of the membrane (iv).

Since amyloid deposits are found in some type II diabetics but not others, its role in type II diabetes was initially ignored. More careful microscopic analysis indicated *β*-cell mass is reduced strongly in islets containing IAPP deposits while neighboring islets lacking the deposits are relatively unaffected [5, 6]. More directly, some forms of IAPP were shown to be toxic to *β*-cells. The relationship between IAPP aggregation and toxicity is not simple as both the freshly dissolved peptide and fully formed fibers show minimal toxicity [7]. Instead, increasing evidence suggests that the toxicity is due to intermediates generated during the assembly of amyloid fibers. These intermediates have been proposed to attack cells in a variety of ways, such as by generating inflammation, creating reactive oxygen species, and overloading the misfolded protein response pathway (Figure 1) [2].

One common, well-studied mechanism of toxicity by IAPP is the disruption of the plasma and organelle membranes [8]. Two of the most commonly studied theories relating to membrane disruption are the pore hypothesis and the fragmentation hypothesis [9–13]. In the pore hypothesis, amyloid species can interact with the membrane surface and oligomerize to form localized pores or channels that cause an uncontrolled, nonphysiological flux of ions across the membrane [9, 10, 14]. In the detergent or fragmentation mechanism, hIAPP intermediates interact with the membrane causing the formation of vesicle-like structures [12, 15, 16]. Membrane fragmentation is due to the process of amyloid formation rather than a property of amyloid fiber itself. In particular, the reactive hydrophobic regions at the ends of amyloid fibrillar may incorporate lipid molecules into the fiber during the ongoing process of aggregation [16]. Supporting this concept, it has been found that while A*β* amyloid fibers and monomeric A*β* are nontoxic by themselves the addition of two species together is strongly toxic to neurons [7, 17]. The two mechanisms of membrane disruption appear to exist simultaneously and the relative balance between each mechanism can be influenced by the cellular environment [18, 19] or ligands [20].

## 2. The IAPP Aggregation Pathway

The IAPP aggregation pathway shows some common characteristics with other amyloidogenic proteins and important differences in other aspects. When freshly dissolved* in vitro*, after complete dissolution of the fiber (at low pH and temperature), the initial dominant species under most conditions is the monomeric peptide [21, 22]. The monomeric hIAPP is primarily unfolded but is not a true statistical random coil. Instead, NMR experiments under these conditions [23, 24] suggest the conformational landscape is biased towards helical conformations, particularly in the N-terminal regions. Similarly, measurements of the hydrodynamic radius by NMR [25] and triplet quenching of hIAPP labeled with tryptophan at the C-terminus [26] suggest a more compact state than expected for an unfolded peptide with the C-terminus folding back to the disulfide bond at the N-terminus [26]. Measurements of the secondary structure of the monomer by circular dichroism (CD) similarly indicate a small amount of secondary structure. Specifically, the NMR, CD, and diffusion measurements indicate monomeric hIAPP adopts a state similar to the premolten globule state in protein folding, with fluctuating metastable structure and condensed fold that is less compact than an unfolded protein but lacking the well-packed core of a natively folded protein [27]. This state appears to be particularly aggregation-prone in general; many other amyloidogenic peptides and proteins also appear to be premolten globules of this type [28–30]. The conformational tendencies of the monomer also appear to be linked to the propensity to aggregate; helix inducing solvents (such as hexafluoroisopropanol (HFIP)) at low concentrations give rise to a dramatic increase in fiber formation rates, although the exact cause of this effect is still not fully understood [31].

Freshly dissolved hIAPP only exists as a monomer under specific conditions. Under the conditions usually used in* in vitro* experiments (neutral pH and temperature 20–37°C), the monomer coexists with a micelle-like aggregate with a CMC of approximately 1.5–2 *μ*M [21]. At lower temperatures and pH 7.3, small oligomers form apparently by N-terminal association while, at pH 5, hIAPP apparently exists exclusively in the monomeric form initially, with very slow self-association [21]. The absence of oligomerization at pH 5 is important because the peptide is initially stored at very high concentrations at low pH in the secretory granule before it is released into the bloodstream and then diluted to lower concentrations [32].

**Figure 2:**
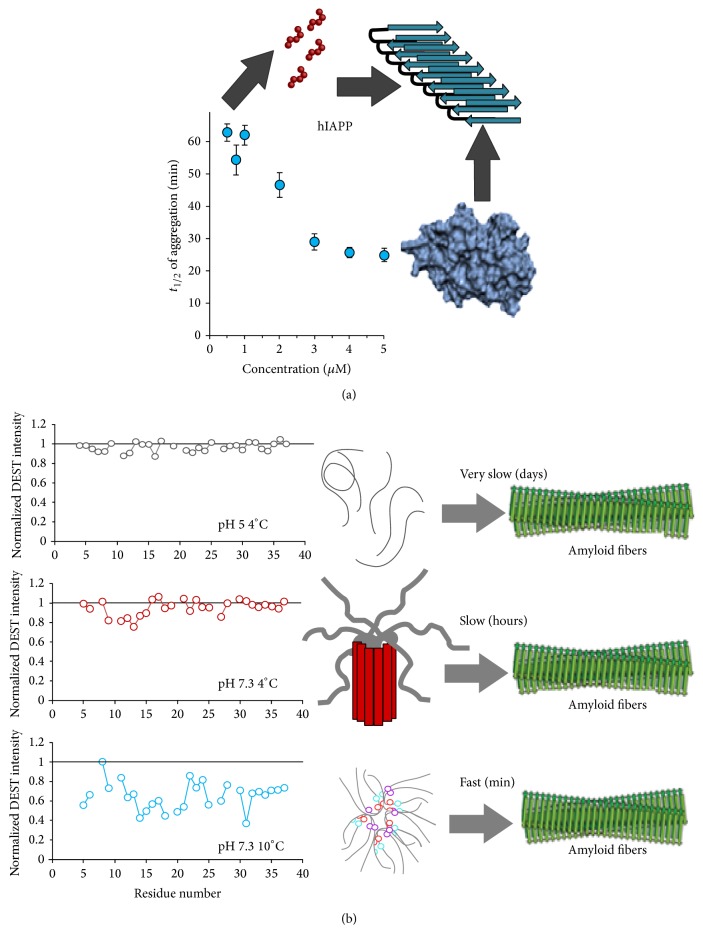
Dependence of IAPP self-association on sample conditions. (a) IAPP aggregation is strongly dependent on the formation of micelles occurring at specific critical micellar concentrations. (b) Mapping of the self-association of hIAPP as a function of pH and temperature. (Top) The micelle is not formed when H18 is ionized and no self-association is observed below pH 6. (Middle) At neutral pH, but below a critical temperature for micelle formation (4°C), small oligomers form which predominantly interact with the N-terminal helix (residues 7–16). (Bottom) Above the temperature for micelle formation (10°C), self-association is found throughout the peptide but particularly in the central region (residues 12–21). Adapted from [21].

In contrast to some other amyloid proteins which form a diverse set of stable or metastable oligomers [33], aggregation by hIAPP appears to be a largely downhill, smooth process under physiological conditions without an appreciable buildup of intermediate aggregation products [22]. Phenomenologically, the aggregation kinetics of hIAPP are sigmoidal with a short lag time followed by explosive fibril growth. The kinetics of IAPP have been successfully modeled by a modified nucleated polymerization mechanism [34]. In this model, aggregation is controlled by the buildup of a small population of energetically unfavorable nuclei that initiate fiber formation. Once fiber formation starts, it is accelerated by a secondary nucleation process caused by fibril breakage. Each broken end of the amyloid fiber serves as a new nucleus for fiber elongation. Micelle formation plays an important role in the aggregation process (Figure 2) [21, 35]. In conditions in which the micelle is present (neutral pH, temperature > 10°C, and concentration > 2 *μ*M), aggregation appears to nucleate within the micelle from the central region of the peptide (residues 12–21) [21]. Under conditions in which the micelle is not present (pH < 6, temperature < 10°C, or concentration < 1 *μ*M), aggregation is much slower and is dominated by contacts within the N-terminus of the peptide [21].

## 3. Targets for Intervention in the Aggregation Pathway

**Figure 3:**
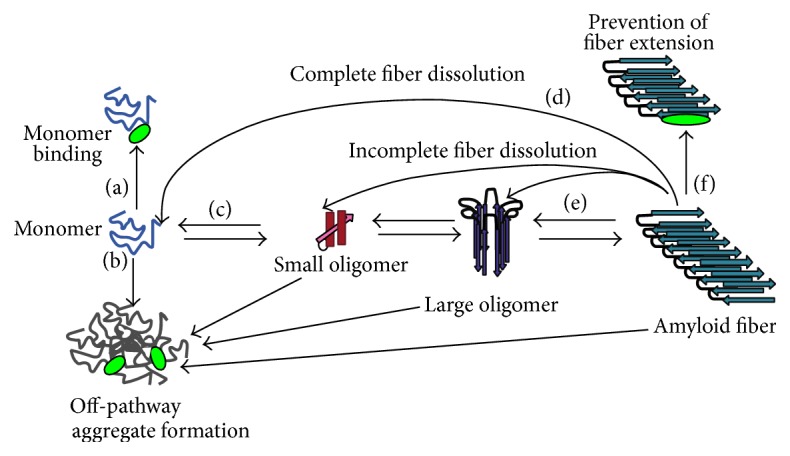
Possible influences of ligands on hIAPP aggregation. Schematic illustrating some of the possible mechanisms by which an inhibitor can affect aggregation: (a) monomer stabilization; (b) stabilization of off-pathway intermediates; (d and e) dissolution of fibers either completely to the monomeric state (d) or incompletely to another oligomeric form (e); (f) prevention of fiber extension. Note that this is not an exhaustive list of possible interactions.

Any point in the aggregation process could conceivably serve as a target for inhibition (Figure 3). An inhibitor could block the formation of toxic oligomers by stabilizing the monomer (Figure 3(a)), diverting the monomeric peptide to off-pathway, nontoxic intermediates (Figure 3(b)), prevent the primary nucleation process by destabilizing small oligomers (Figure 3(c)), prevent fiber elongation and stop secondary nucleation by capping reactive fiber ends (Figure 3(f)), or destabilize the fiber into either monomers (Figure 2(d)) or other oligomeric species (Figure 3(e)). Note that diverting aggregation is not the same as stopping toxicity; in some cases the oligomers formed by dissolution of the fiber are even more toxic generated in the unperturbed aggregation process.

Given this complexity, identifying the actual target of inhibition is challenging. Amyloid fibers are commonly detected by the fluorescent dye thioflavin T (ThT), which becomes fluorescent when bound to the cross-*β* spine of the amyloid fiber [36]. This method has become dominant in amyloid research as, unlike many other techniques, it lends itself naturally to high-throughput analysis through multiwell plates, allowing the real time characterization of the kinetics of aggregation under multiple conditions simultaneously with multiple inhibitors. Since the intensity of the fluorescent signal is believed to be proportional to the concentration of fibers present, a shift in the time constant of the sigmoid upon the addition of a molecule is often interpreted as inhibition of fiber formation by the molecule. More specifically, an increase in the lag time is usually interpreted in terms of the nucleated polymerization model as inhibition of nucleation (Figures 3(a) or 3(c)). Analogously, a decrease in the final intensity at equilibrium when an inhibitor is added is sometimes interpreted as a shift of the equilibrium constant away from fiber formation towards other, nonfibrillar species. A decrease in the slope of the sigmoid can be interpreted within this model as inhibition of fiber elongation (Figure 3(f)). The reverse reaction, adding the putative inhibitor to fully formed fibers, and the seeding reaction, adding monomer to fully formed fibers, can help establish if the inhibitor can destabilize the fiber (Figures 3(d) and 3(e)) or blocks reactive fiber ends (Figure 3(f)).

The ambiguity in these statements is deliberate and reflects the actual ambiguity in interpreting ThT results. Small molecule inhibitors such as curcumin and quercetin that overlap in absorbance at the excitation wavelength of ThT can yield a false positive for fibril inhibition, as the fluorescence of ThT is decreased by an inner-filter effect [37]. More commonly, the putative inhibitor may bind to the same site as ThT on the amyloid fiber, resulting in a false positive for inhibition from displacement of ThT by competitive inhibition [38]. Alternatively, ThT (Kd ~ 1 *μ*M) [39] may shield a weakly binding inhibitor from binding to the fiber, yielding a false negative result [22]. Finally, ThT is only sensitive to amyloid fiber formation and cannot distinguish the different oligomeric species that may arise.

A more rigorous approach is to directly observe fibers by microscopy. However, the technique is low-throughput and prone to experimental bias both due to inadequate sampling from uneven distribution on the substrate and the uneven affinity of different oligomers for the substrate surface [40]. A promising approach is ligand based detection through saturation transfer experiments, which not only directly establish binding to large oligomeric species but also provide information on intermolecular contacts [41, 42]. Unlike thioflavin T experiments, saturation transfer difference (STD) NMR spectroscopy is not limited to the amyloid fiber alone but can measure the interaction of a ligand with any species large enough to enable the observation of spin diffusion from the oligomer to the ligand. Importantly, it is one of the few techniques that can give the specific information on intermolecular contacts important for optimization of the ligand.

## 4. Natural Products Are Natural Choices for Amyloid Intervention

Particular areas of interest toward discovering agents against amyloidosis have been natural products. Natural products are small molecules found abundantly in nature, particularly in foods and have been the main source for early medicines and therapeutics. They feature specific scaffolds that have made them beneficial receptor agonists, enzyme activators, inhibitors of protein-protein and DNA-protein interactions and channel openers [43]. Some natural products have also been shown to act as colloidal species which can sequester low molecular weight aggregates and prevent their fibrillation [44]. Importantly, natural products often exhibit better pharmacological profiles than their synthetic counterparts, especially with regard to toxicity and absorption [43]. Based on early successes, natural products such as flavonoids and curcuminoids have been extensively researched in regard to reduction of the amyloid associated toxicity of A*β* and *α*-synuclein.

**Figure 4:**
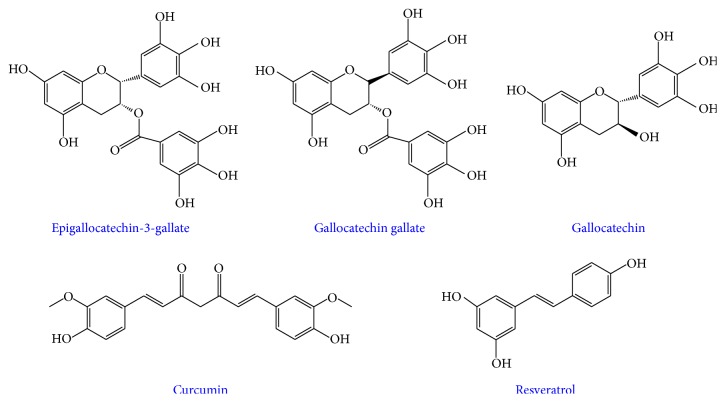
Chemical structures of the most studied polyphenolic small molecule inhibitors of hIAPP aggregation: epigallocatechin gallate (EGCG), gallocatechin gallate (GCG), gallocatechin (GC), curcumin, and resveratrol.

There have been two main approaches to blocking the toxicity associated with amyloid aggregation. The first approach is to reduce toxicity by preventing the toxic species from forming. The second approach attempts to mediate the effects of the toxic oligomer formation by serving as an antioxidant to reduce the reactive oxygen species (ROS) generated by the aggregation process [45, 46], reduce inflammatory effects [47], prevent membrane association [48, 49], or block the channels created by the peptide [50–53]. Given their ability to target multiple facets of amyloid associated toxicity through their anti-inflammatory, antioxidant, and antiamyloidogenic properties [54, 55], natural products make a very promising class of candidates as viable small molecule inhibitors toward amyloids, specifically hIAPP. Through more stringent investigations, the use of natural products as aggregation modulators of hIAPP has provided more direct structural information about the hIAPP aggregation process itself. Herein, we will discuss the application of small molecule natural products toward the modulation of hIAPP aggregation with a particular focus on the most studied natural products, epigallocatechin-3-gallate (EGCG), curcumin, and resveratrol (Figure 4).

## 5. EGCG Diverts hIAPP to Nontoxic Off-Pathway Oligomers

EGCG is a naturally occurring flavonoid extracted from green tea that has shown promise as a possible therapeutic for amyloid related diseases, entering early clinical trials for the prevention of Alzheimer's, Parkinson's, and Huntington's disease and is currently one of the best studied natural products against amyloid aggregation [56]. EGCG has been shown* in vitro* to redirect the aggregation pathway of multiple amyloids to form off-pathway, amorphous aggregates with minimal toxicity [54, 57–60]. At substoichiometric levels, the intensity of ThT fluorescence decreases and the lag phase lengthens when EGCG is added at the start of the aggregation process, suggesting hIAPP is still able to form *β*-sheet containing aggregates but at a reduced rate [59, 60]. However, when stoichiometric to excess amounts of EGCG are added in solution, a complete mitigation of the ThT fluorescence signal is observed. NMR experiments indicate that EGCG may compete with ThT for fibril binding depending on the relative concentrations of EGCG and ThT; however, under normal ratios of EGCG and ThT, EGCG diverts aggregation through a fast consumption of hIAPP monomer to a larger nonfibrillar aggregate which EGCG may interact with [22]. These results were also confirmed by electron microscopy where more small, amorphous aggregates were observed when hIAPP is coincubated with EGCG with a more pronounced decrease as the incubation time was increased [22].

The addition of EGCG at various points along the aggregation pathway also modifies the structural characteristics of the aggregates formed depending on which point EGCG is added during the aggregation process. When it is added during the middle and end of the lag phase, small nonfibrillar aggregates with some amorphous content were formed. When it is added to early nucleation phase, the preformed *β*-sheet containing species were remolded to smaller, thinner aggregates which have minimal *β*-sheet character, demonstrating that EGCG may not reverse fibrillation to monomer, but to some other low molecular weight species [22, 59–61]. Finally, when EGCG was added to preformed amyloid fibers the ThT signal did not reach the baseline and electron micrographs indicate that the nonfibrillar aggregates formed from the dissolution of hIAPP fibers were morphologically distinct from the aggregates formed from the forward reaction of hIAPP in the presence of EGCG [60]. Seeding experiments shed light onto a possible mechanism of assembly by displaying whether or not a fibril seed formed in the presence of EGCG can promote aggregation of the peptide from a monomeric state. When a fibril seed is added to hIAPP monomer in solution without EGCG, the peptide aggregates are almost immediately converted to amyloid fibrils with a very short lag phase. However, when a seed of a 1 : 1 EGCG : hIAPP sample was added to a freshly dissolved sample of hIAPP, the aggregation profile was not affected, suggesting that the seed may still promote fibrillation through the natural pathway even in the presence of EGCG [59].

Recently, ion mobility mass spectrometry studies were utilized to probe possible mechanisms for inhibition as well as disaggregation of EGCG and hIAPP. EGCG was shown to bind to the monomer (up to 3 molecules of EGCG), which induces a conformational change that inhibits the formation of higher order species in a dose dependent manner through hydrophobic interactions which was also verified through ThT and TEM studies [61]. These investigations also gave insight into a possible mode of disaggregation of hIAPP. In the presence of varying concentrations of EGCG, the small molecule depolymerizes preformed aggregates to a more amorphous structure which forms with a low monomer population [61].

At the molecular level, EGCG was initially proposed to interfere with aromatic *π*-*π* stacking interactions in the amyloid fiber as a general mechanism for amyloid inhibition [54]. In support of this mechanism, studies with a modified version of EGCG lacking the gallate ester (GC) displayed noticeably less inhibition verifying that the trihydroxyl phenyl rings are important for hIAPP inhibition possibly through hydrophobic and H-bond contacts [59]. However, EGCG still displayed inhibitory properties similar to wild-type hIAPP in an IAPP mutant in which the aromatic residues were changed to leucine, which suggests that the *π*-*π* interactions may not be essential for attenuation of aggregation [59]. EGCG inhibited fibril formation in truncated peptide variants (8–37) with an acetylated and free amine N-terminus indicating that the N-terminus and Cys residues may not be particularly important in interacting with EGCG. EGCG oxidizes readily in solution and the oxidized forms can covalently link to free amines and, if the disulfide bond is reduced, to cysteine residues in IAPP through Schiff base formation [58, 62]. However, when an oxidized version of EGCG was used to study inhibition, the small molecule also demonstrated inhibitory properties independent of covalent binding to IAPP; thus the Schiff base may not be critical for inhibition, suggesting a possible hydrophobic mechanism for reforming aggregates. The role for hydrophobic interactions is partially borne out by experiments with EGCG and hIAPP in the presence of a phospholipid monolayer. They found that the interaction of the peptide and the lipid stabilizes IAPP at the membrane surface [63]. These contacts may prevent EGCG from accessing hydrophobic binding sites on the peptide and reduce its ability to both inhibit aggregation and remodel preformed aggregates.

Simulation studies of EGCG with the 5-mer and 10-mer oligomers have identified that *π*-*π* stacking and H-bond interactions are possible avenues of binding to these species with specific contacts with residues 23, 25, 26, 27, and 37 [64]. This result conflicts with reports that EGCG can inhibit the hIAPP-8-24 fragment and a modified hIAPP without aromatic residues, suggesting multiple mechanisms may exist with different efficacies depending on the actual contacts present.

Taken together, these experiments indicate EGCG modifies hIAPP aggregation by a complex process that is not the stabilization of the monomeric species or complete dissolution of the fiber into monomers. EGCG does not stabilize monomers since NMR studies revealed that monomers disappear during aggregation and oligomeric species can be detected by microscopy. However, the mechanism is not strictly thermodynamic stabilization of off-pathway oligomers, since adding EGCG to preformed fibers results in smaller *β*-sheet fibers rather than the amorphous aggregates seen if EGCG is added at the beginning of the aggregation process. In this case, it would be expected that addition of EGCG at any time-point would provide similar results.

## 6. Curcumin Destabilizes Helical Intermediates of hIAPP

Similar to EGCG, curcumin, a natural product found abundantly in turmeric has been widely used as a therapeutic due to its antioxidant, anticancer, antibiotic, and antiamyloidogenic properties. It has been shown to nonspecifically bind to amyloid-*β* and modify its aggregation pathway; however less is known about the interactions between curcumin and hIAPP. Curcumin has demonstrated inhibitory properties against hIAPP aggregation; however, its mechanism of action still remains elusive. Studying the effects of curcumin on amyloid aggregation can be difficult by conventional fluorescence methods due to its ability to displace ThT and its strong absorption in the excitation range of ThT [37]; therefore other biophysical and spectroscopic techniques have been used to monitor the inhibitory activity of curcumin. Furthermore, curcumin is insoluble in water and its stability is dependent on sample conditions and therefore studying its role with peptides must be completed carefully.

The ability of curcumin to suppress the formation of mature fibrils has been observed through the retainment of NMR signal intensity of the amide peaks in the presence of curcumin, which was significantly reduced in the absence of the small molecule [65]. Intensities of cross-peaks observed in the 2D ^1^H/^1^H TOCSY spectrum did not change in the presence of curcumin, confirming the propensity of the small molecule to mitigate fibril formation. The TEM images of samples at the end time-point (45 days) showed morphology markedly different than when curcumin is not present [66]. The micrographs showed smaller species which do not resemble traditional amyloid fibrils. When curcumin was added to a sample of peptide containing oligomeric intermediates with helical structure, the secondary structure content shifted to more random coil assemblies, suggesting that curcumin may disfavor helical oligomers which can promote fibrillation [65].

## 7. Resveratrol Blocks hIAPP Membrane Association

The third natural product to be discussed in this review has been studied to a lesser extent than EGCG. Resveratrol is a polyphenolic stilbene derivative found mainly in grapes and red wine. Resveratrol has been shown to inhibit the fibrillation of A*β* and reduce inflammation as well as reduce the amount of intracellular A*β* levels [45]. However, little is known regarding how resveratrol interacts with hIAPP. The small molecule has demonstrated to have inhibitory activity against hIAPP (IC_50_ = 3.3 *μ*M) and reduce hIAPP fibrillation by diverting aggregation to an off-pathway species [48]. Due to the interference of resveratrol with ThT, it has been difficult to study the aggregation kinetics by fluorescence assays. Other biophysical and analytical methods have been employed in order to probe the relationship between hIAPP and resveratrol more closely [48, 67]. Early investigations measured IAPP species by AFM which displayed small, spherical aggregates as opposed to fibrillar species. Transmission electron micrographs showed that even at a large excess of resveratrol, IAPP fibril formation was not completely abolished, as seen with EGCG. The lag phase was increased; however, smaller, thinner fibrillar species were observed. ESI mass spectrometry probed that there is no strong interaction between monomeric hIAPP and excess resveratrol, suggesting there is a weak interaction between these two, also suggested by MD simulation studies which indicated a possible *β*-sheet U-shaped confirmation that may block hIAPP from interacting with other *β*-sheet monomers [68, 69]. Early investigations by NMR studies showed chemical shift changes in H18 and K1 upon titration with resveratrol revealing these residues may be involved in interacting with resveratrol [70]. A detailed study investigated the effects of hIAPP mutations and how they perturb resveratrol binding and inhibitory activity. These were specifically designed to measure the involvement of *π*-cation interactions, hydrophobic stacking through aromatic residues, and the N-terminus. The results verified that H18 and R11 may have played a role in interacting with resveratrol, possibly through *π*-cation interactions; however a lesser but still visible effect by aromatic *π*-*π* stacking may not be a primary mode of interaction between small molecule and peptide as hypothesized before [67]. This study also examined the ability for resveratrol to remodel hIAPP fibrils to smaller species, but unlike EGCG no substantial modulation of preformed fibrils was seen [67].

Studies of how resveratrol may impact aggregation and membrane disruption in the presence of hIAPP have also been conducted, which may shed light into possible mechanisms towards alleviating membrane-induced toxicity [48, 49, 68, 71]. The aggregation of hIAPP occurs at a faster rate in the presence of negatively charged lipid bilayers, which may be responsible for membrane-induced toxicity. When resveratrol was coincubated with hIAPP and PC/PG vesicles, peptide aggregation was inhibited as seen by FTIR [48]. A possible mechanism has been proposed based on X-ray synchrotron studies suggesting that resveratrol first inserts itself in the upper chain region and decreases the chain packing of the lipids [49]. However, the insertion is incomplete and a substantial fraction of resveratrol remains in solution. The soluble resveratrol fraction was proposed to interact strongly with exposed hydrophobic interfaces in soluble hIAPP, masking these sites and preventing the hIAPP from interacting with the bilayer [49]. In this way, the structural integrity of the membrane is maintained in the presence of hIAPP [49]. The membrane-resveratrol-hIAPP system has also been investigated by simulation studies, which suggest resveratrol may inhibit hIAPP fibrillation at the membrane surface by locking the peptide confirmation into a helix and thus not allowing the structural transitions required for further aggregation [68].

**Figure 5:**
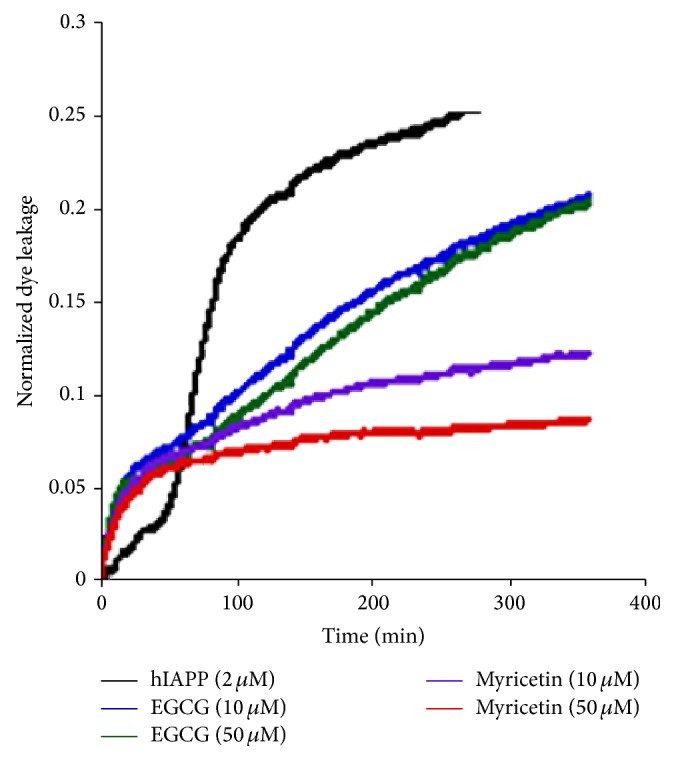
Dye leakage induced upon hIAPP addition (2 *μ*M) to 0.2 mg/mL POPC/POPG (7 : 3 molar ratio) lipid vesicles in the presence and absence of indicated flavanols. Membrane disruption is detected by concentration dependent quenching of a vesicle-encapsulated dye. Within the vesicle, the carboxyfluorescein dye is at a high concentration and is quenched. Disruption of the membrane integrity of the vesicle dilutes the concentration of the dye and thereby increases the measured fluorescence.

**Figure 6:**
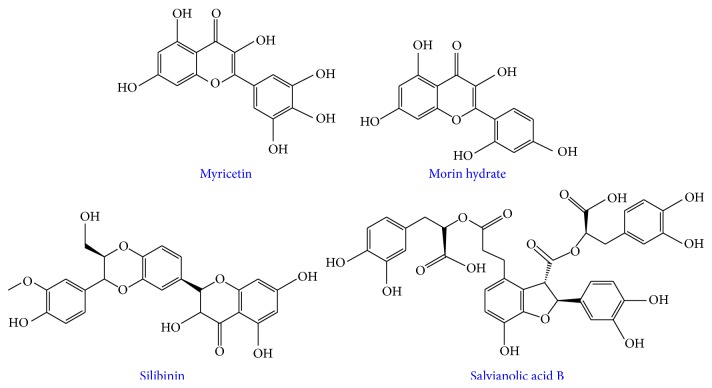
Chemical structures of other polyphenolic small molecule inhibitors of hIAPP aggregation.

Lastly, toxicity studies indicated that, in the presence of resveratrol, INS-1 cells had 90% viability, demonstrating its positive effects on cells under the influence of hIAPP. Interestingly, the relative effectiveness of EGCG and resveratrol in protecting aggregation induced membrane damage is opposite in hIAPP (resveratrol > EGCG) compared to the amyloidogenic protein *β*-microglobulin (EGCG > resveratrol) [72], suggesting interaction rules are not completely general for all amyloids and further that efficacy against aggregation in the solution phase is not a guarantee of efficacy against membrane mediated aggregation or pore formation. Along these lines, we measured the ability of several flavanols to inhibit membrane disruption by hIAPP. In this experiment, membrane disruption typically occurs in two stages. The first stage is reflective of early pore formation while the second stage is reflective of a large-scale detergent-like disruption caused by the incorporation of membrane lipids into the growing fiber [18–20, 73, 74]. Notably, stoichiometric amounts of EGCG and another flavanol myricetin (see Figure 6 for structure) enhanced early pore formation (first 50 minutes, Figure 5) while suppressing the latter phase associated with fibril growth on the membrane (Figure 5), a behavior similar to insulin, a strong inhibitor of hIAPP fiber formation [20].

## 8. Other Flavanols

EGCG, curcumin, and resveratrol have been the most widely studied natural products with hIAPP; however, other naturally occurring compounds may also be useful toward probing, modulating, and understanding IAPP aggregation. Myricetin has demonstrated to have antiamyloidogenic properties against A*β*; however, its effects were less clear with hIAPP. AFM studies indicated that myricetin in high molar excess prevented the formation of insoluble fibrillar species [75]; however by TEM, even in the presence of myricetin, thin fibrillar aggregates were present in solution [76]. Cell viability assays also indicated that there was partial rescue of cell viability in the presence of myricetin, compared to when only hIAPP was added. Similarly, inositol compounds, which have shown promise in inhibiting A*β* aggregation, are ineffective against hIAPP [77]. On the other hand, another polyphenolic compound, morin hydrate, showed some promise toward modifying hIAPP aggregation [76]. When incubated with hIAPP, in excess, morin hydrate formed small, thin IAPP species with some amorphous character. Similarly, morin hydrate showed the propensity to disaggregate preformed aggregates when added stoichiometrically [76]. Morin hydrate differs from myricetin in its B-ring substitution, which may influence its role in modifying hIAPP aggregation and therefore can be used another framework for modulating aggregation.

Some promising data testing silibinin, a natural product extracted from the seeds of the herb milk thistle, against hIAPP has shown potential for this class of molecules to be used as possible inhibitors and aggregation modulators [61]. Silibinin favored the 3+ monomer as seen by mass spectrometry and does not allow oligomerization of the peptide. Electron microscopy also showed that no fibrillar species were present when incubated with excess silibinin [61]. This natural product can also disaggregate preformed fibrils into smaller oligomeric assemblies, which may be nontoxic, off-pathway oligomers. Similarly, salvianolic acid B was found to inhibit both the formation of fibers and lower order oligomers as well as suppress membrane disruption and cytotoxicity [78].

## 9. Conclusions

Natural products have shown some promise against hIAPP aggregation and hIAPP induced toxicity but there is still much to be done and many fundamental questions remain unanswered. Before much progress can be made, it is essential to know the actual dominant mechanism of toxicity by hIAPP. It is also essential that methods can be developed for high-throughput screening for potential lead compounds [79–82]. However, most of these assays currently select against fiber inhibition with the assumption that stopping fiber formation will stop hIAPP toxicity. Alternatively, the dye leakage assay can be adapted to a high-throughput format for screening compound libraries for the ability to attenuate membrane disruption (Figure 5) [83–85]. Less studied is how other potential mechanisms may contribute to toxicity. IAPP is known to bind copper [86–88] and there are conflicting reports on whether metal association can drive the formation of reactive oxygen species similar to the A*β* peptide [87, 88]. Natural products often exhibit antioxidative properties that may reduce ROS and may open up a new potential avenue for treatment.

## 10. Future Directions

*In vitro* biochemical and biophysical assays have provided a basis for more clearly understanding how small molecule natural products influence hIAPP aggregation and toxicity. Recently, mass spectrometry has been employed to give more detailed information about specific interactions between these molecules and their peptide target [61, 89]; however to probe these interactions further, atomic-level resolution techniques need to be utilized. The screening of natural products using high-resolution NMR methods has not yet been used for studying hIAPP but has demonstrated to be effective for other amyloid proteins. Measuring peak intensities and broadening through simple 1D NMR experiments can provide valuable information about the effect of ligands on hIAPP [65, 66]. Furthermore, STD NMR experiments have been used as a method for monitoring the contacts between specific atoms of a ligand and a larger protein target, like amyloid-*β* [90]. Small molecules can also be screened against amyloid proteins using two-dimensional techniques, such as Heteronuclear Multiple Quantum Coherence (HMQC). Using these techniques it is possible to directly obtain residue specific information of ligand interactions with the peptide using a fast data acquisition approach (SOFAST) [91], which can circumvent the possible changes in chemical shifts associated with peptide aggregation rather than ligand binding [92]. Solid-state NMR methods can also be employed to investigate structural features related to small molecule interactions with amyloids when inaccessible to solution techniques, as seen with A*β* and EGCG [93] and curcumin and resveratrol [94]. Overall, NMR can be an effective tool to study the interactions between natural products and hIAPP, providing atomic-level details unobtainable by other techniques which can help elucidate mechanisms of inhibition or oligomer formation and stabilization.

## References

[1] Rushing P. A., Hagan M. M., Seeley R. J., Lutz T. A., Woods S. C. (2000). Amylin: a novel action in the brain to reduce body weight. *Endocrinology*.

[2] Westermark P., Andersson A., Westermark G. T. (2011). Islet amyloid polypeptide, islet amyloid, and diabetes mellitus. *Physiological Reviews*.

[3] Opie E. L. (1901). On the relation of chronic interstitial pancreatitis to the islands of Langerhans and to diabetes melutus. *Journal of Experimental Medicine*.

[4] Westermark P., Wernstedt C., Wilander E., Sletten K. (1986). A novel peptide in the calcitonin gene related peptide family as an amyloid fibril protein in the endocrine pancreas. *Biochemical and Biophysical Research Communications*.

[5] Clark A., Wells C. A., Buley I. D. (1988). Islet amyloid, increased A-cells, reduced B-cells and exocrine fibrosis: quantitative changes in the pancreas in type 2 diabetes. *Diabetes Research*.

[6] Westermark P., Wilander E. (1978). The influence of amyloid deposits on the islet volume in maturity onset diabetes mellitus. *Diabetologia*.

[7] Schlamadinger D. E., Miranker A. D. (2014). Fiber-dependent and -independent toxicity of islet amyloid polypeptide. *Biophysical Journal*.

[8] Lashuel H. A. (2005). Membrane permeabilization: a common mechanism in protein-misfolding diseases. *Science of Aging Knowledge Environment*.

[9] Quist A., Doudevski I., Lin H. (2005). Amyloid ion channels: a common structural link for protein-misfolding disease. *Proceedings of the National Academy of Sciences of the United States of America*.

[10] Mirzabekov T. A., Lin M.-C., Kagan B. L. (1996). Pore formation by the cytotoxic islet amyloid peptide amylin. *The Journal of Biological Chemistry*.

[11] Green J. D., Kreplak L., Goldsbury C. (2004). Atomic force microscopy reveals defects within mica supported lipid bilayers induced by the amyloidogenic human amylin peptide. *Journal of Molecular Biology*.

[12] Engel M. F. M., Khemtémourian L., Kleijer C. C. (2008). Membrane damage by human islet amyloid polypeptide through fibril growth at the membrane. *Proceedings of the National Academy of Sciences of the United States of America*.

[13] Capone R., Quiroz F. G., Prangkio P. (2009). Amyloid-*β*-induced ion flux in artificial lipid bilayers and neuronal cells: resolving a controversy. *Neurotoxicity Research*.

[14] Last N. B., Miranker A. D. (2013). Common mechanism unites membrane poration by amyloid and antimicrobial peptides. *Proceedings of the National Academy of Sciences of the United States of America*.

[15] Milanesi L., Sheynis T., Xue W.-F. (2012). Direct three-dimensional visualization of membrane disruption by amyloid fibrils. *Proceedings of the National Academy of Sciences of the United States of America*.

[16] Brender J. R., Dürr U. H. N., Heyl D., Budarapu M. B., Ramamoorthy A. (2007). Membrane fragmentation by an amyloidogenic fragment of human islet amyloid polypeptide detected by solid-state NMR spectroscopy of membrane nanotubes. *Biochimica et Biophysica Acta*.

[17] Jan A., Adolfsson O., Allaman I. (2011). A*β*42 neurotoxicity is mediated by ongoing nucleated polymerization process rather than by discrete A*β*42 species. *The Journal of Biological Chemistry*.

[18] Sciacca M. F. M., Brender J. R., Lee D.-K., Ramamoorthy A. (2012). Phosphatidylethanolamine enhances amyloid fiber-dependent membrane fragmentation. *Biochemistry*.

[19] Sciacca M. F. M., Milardi D., Messina G. M. L. (2013). Cations as switches of amyloid-mediated membrane disruption mechanisms: calcium and IAPP. *Biophysical Journal*.

[20] Brender J. R., Lee E. L., Hartman K. (2011). Biphasic effects of insulin on islet amyloid polypeptide membrane disruption. *Biophysical Journal*.

[21] Brender J. R., Krishnamoorthy J., Sciacca M. F. (2015). Probing the sources of the apparent irreproducibility of amyloid formation: drastic changes in kinetics and a switch in mechanism due to micellelike oligomer formation at critical concentrations of IAPP. *The Journal of Physical Chemistry B*.

[22] Suzuki Y., Brender J. R., Hartman K., Ramamoorthy A., Marsh E. N. G. (2012). Alternative pathways of human islet amyloid polypeptide aggregation distinguished by ^19^F nuclear magnetic resonance-detected kinetics of monomer consumption. *Biochemistry*.

[23] Williamson J. A., Miranker A. D. (2007). Direct detection of transient *α*-helical states in islet amyloid polypeptide. *Protein Science*.

[24] Yonemoto I. T., Kroon G. J. A., Dyson H. J., Balch W. E., Kelly J. W. (2008). Amylin proprotein processing generates progressively more amyloidogenic peptides that initially sample the helical state. *Biochemistry*.

[25] Soong R., Brender J. R., Macdonald P. M., Ramamoorthy A. (2009). Association of highly compact type II diabetes related islet amyloid polypeptide intermediate species at physiological temperature revealed by diffusion NMR spectroscopy. *Journal of the American Chemical Society*.

[26] Vaiana S. M., Best R. B., Yau W.-M., Eaton W. A., Hofrichter J. (2009). Evidence for a partially structured state of the amylin monomer. *Biophysical Journal*.

[27] Chaffotte A. F., Iñaki Guijarro J., Guillou Y., Delepierre M., Goldberg M. E. (1997). The ‘pre-molten globule,’ a new intermediate in protein folding. *Journal of Protein Chemistry*.

[28] Uversky V. N., Fink A. L. (2004). Conformational constraints for amyloid fibrillation: the importance of being unfolded. *Biochimica et Biophysica Acta—Proteins and Proteomics*.

[29] Brender J. R., Nanga R. P. R., Popovych N., Soong R., Macdonald P. M., Ramamoorthy A. (2011). The amyloidogenic SEVI precursor, PAP248-286, is highly unfolded in solution despite an underlying helical tendency. *Biochimica et Biophysica Acta—Biomembranes*.

[30] Vivekanandan S., Brender J. R., Lee S. Y., Ramamoorthy A. (2011). A partially folded structure of amyloid-beta(1-40) in an aqueous environment. *Biochemical and Biophysical Research Communications*.

[31] Yanagi K., Ashizaki M., Yagi H., Sakurai K., Lee Y.-H., Goto Y. (2011). Hexafluoroisopropanol induces amyloid fibrils of islet amyloid polypeptide by enhancing both hydrophobic and electrostatic interactions. *The Journal of Biological Chemistry*.

[32] Hutton J. C. (1982). The internal pH and membrane potential of the insulin-secretory granule. *Biochemical Journal*.

[33] Suzuki Y., Brender J. R., Soper M. T. (2013). Resolution of oligomeric species during the aggregation of A*β*
_1−40_ using ^19^F NMR. *Biochemistry*.

[34] Padrick S. B., Miranker A. D. (2002). Islet amyloid: phase partitioning and secondary nucleation are central to the mechanism of fibrillogenesis. *Biochemistry*.

[35] Rhoades E., Gafni A. (2003). Micelle formation by a fragment of human islet amyloid polypeptide. *Biophysical Journal*.

[36] Biancalana M., Koide S. (2010). Molecular mechanism of thioflavin-T binding to amyloid fibrils. *Biochimica et Biophysica Acta—Proteins and Proteomics*.

[37] Hudson S. A., Ecroyd H., Kee T. W., Carver J. A. (2009). The thioflavin T fluorescence assay for amyloid fibril detection can be biased by the presence of exogenous compounds. *The FEBS Journal*.

[38] Meng F., Marek P., Potter K. J., Verchere C. B., Raleigh D. P. (2008). Rifampicin does not prevent amyloid fibril formation by human islet amyloid polypeptide but does inhibit fibril thioflavin-T interactions: implications for mechanistic studies of *β*-cell death. *Biochemistry*.

[39] Lockhart A., Ye L., Judd D. B. (2005). Evidence for the presence of three distinct binding sites for the thioflavin T class of Alzheimer's disease PET imaging agents on *β*-amyloid peptide fibrils. *The Journal of Biological Chemistry*.

[40] Hepler R. W., Grimm K. M., Nahas D. D. (2006). Solution state characterization of amyloid *β*-derived diffusible ligands. *Biochemistry*.

[41] DeToma A. S., Krishnamoorthy J., Nam Y. (2014). Synthetic flavonoids, aminoisoflavones: interaction and reactivity with metal-free and metal-associated amyloid-beta species. *Chemical Science*.

[42] Lee S., Zheng X., Krishnamoorthy J. (2014). Rational design of a structural framework with potential use to develop chemical reagents that target and modulate multiple facets of Alzheimer's disease. *Journal of the American Chemical Society*.

[43] Beghyn T., Deprez-Poulain R., Willand N., Folleas B., Deprez B. (2008). Natural compounds: leads or ideas? Bioinspired molecules for drug discovery. *Chemical Biology and Drug Design*.

[44] Blanchard B. J., Chen A., Rozeboom L. M., Stafford K. A., Weigele P., Ingram V. M. (2004). Efficient reversal of Alzheimer's disease fibril formation and elimination of neurotoxicity by a small molecule. *Proceedings of the National Academy of Sciences of the United States of America*.

[45] Jang J. H., Surh Y. J. (2003). Protective effect of resveratrol on beta-amyloid-induced oxidative PC12 cell death. *Free Radical Biology and Medicine*.

[46] Chakrabarti S., Sinha M., Thakurta I. G., Banerjee P., Chattopadhyay M. (2013). Oxidative stress and amyloid beta toxicity in Alzheimer's disease: intervention in a complex relationship by antioxidants. *Current Medicinal Chemistry*.

[47] Apetz N., Munch G., Govindaraghavan S., Gyengesi E. (2014). Natural compounds and plant extracts as therapeutics against chronic inflammation in Alzheimer's disease—a translational perspective. *CNS Neurological Disorders—Drug Targets*.

[48] Mishra R., Sellin D., Radovan D., Gohlke A., Winter R. (2009). Inhibiting islet amyloid polypeptide fibril formation by the red wine compound resveratrol. *ChemBioChem*.

[49] Evers F., Jeworrek C., Tiemeyer S. (2009). Elucidating the mechanism of lipid membrane-induced IAPP fibrillogenesis and its inhibition by the red wine compound resveratrol: a synchrotron X-ray reflectivity study. *Journal of the American Chemical Society*.

[50] Arispe N., Diaz J., Durell S. R., Shafrir Y., Guy H. R. (2010). Polyhistidine peptide inhibitor of the A*β* calcium channel potently blocks the A*β*-induced calcium response in cells. Theoretical modeling suggests a cooperative binding process. *Biochemistry*.

[51] Arispe N., Diaz J. C., Simakova O. (2007). A*β* ion channels. Prospects for treating Alzheimer's disease with A*β* channel blockers. *Biochimica et Biophysica Acta*.

[52] Diaz J. C., Simakova O., Jacobson K. A., Arispe N., Pollard H. B. (2009). Small molecule blockers of the Alzheimer Abeta calcium channel potently protect neurons from Abeta cytotoxicity. *Proceedings of the National Academy of Sciences of the United States of America*.

[53] Fantini J., Di Scala C., Yahi N. (2014). Bexarotene blocks calcium-permeable ion channels formed by neurotoxic Alzheimer's *β*-amyloid peptides. *ACS Chemical Neuroscience*.

[54] Porat Y., Abramowitz A., Gazit E. (2006). Inhibition of amyloid fibril formation by polyphenols: structural similarity and aromatic interactions as a common inhibition mechanism. *Chemical Biology and Drug Design*.

[55] Stefani M., Rigacci S. (2013). Protein folding and aggregation into amyloid: the interference by natural phenolic compounds. *International Journal of Molecular Sciences*.

[56] Mähler A., Mandel S., Lorenz M. (2013). Epigallocatechin-3-gallate: a useful, effective and safe clinical approach for targeted prevention and individualised treatment of neurological diseases?. *EPMA Journal*.

[57] Ehrnhoefer D. E., Bieschke J., Boeddrich A. (2008). EGCG redirects amyloidogenic polypeptides into unstructured, off-pathway oligomers. *Nature Structural & Molecular Biology*.

[58] Palhano F. L., Lee J., Grimster N. P., Kelly J. W. (2013). Toward the molecular mechanism(s) by which EGCG treatment remodels mature amyloid fibrils. *Journal of the American Chemical Society*.

[59] Cao P., Raleigh D. P. (2012). Analysis of the inhibition and remodeling of islet amyloid polypeptide amyloid fibers by flavanols. *Biochemistry*.

[60] Meng F., Abedini A., Plesner A., Verchere C. B., Raleigh D. P. (2010). The Flavanol (-)-epigallocatechin 3-gallate inhibits amyloid formation by islet amyloid polypeptide, disaggregates amyloid fibrils, and protects cultured cells against IAPP-induced toxicity. *Biochemistry*.

[61] Young L. M., Saunders J. C., Mahood R. A. (2014). Screening and classifying small-molecule inhibitors of amyloid formation using ion mobility spectrometry–mass spectrometry. *Nature Chemistry*.

[62] Popovych N., Brender J. R., Soong R. (2012). Site specific interaction of the polyphenol EGCG with the SEVI amyloid precursor peptide PAP(248–286). *The Journal of Physical Chemistry B*.

[63] Engel M. F. M., Vandenakker C. C., Schleeger M., Velikov K. P., Koenderink G. H., Bonn M. (2012). The polyphenol EGCG inhibits amyloid formation less efficiently at phospholipid interfaces than in bulk solution. *Journal of the American Chemical Society*.

[64] Wang Q., Guo J., Jiao P., Liu H., Yao X. (2014). Exploring the influence of EGCG on the *β*-sheet-rich oligomers of human islet amyloid polypeptide (hIAPP1-37) and identifying its possible binding sites from molecular dynamics simulation. *PloS ONE*.

[65] Sparks S., Liu G., Robbins K. J., Lazo N. D. (2012). Curcumin modulates the self-assembly of the islet amyloid polypeptide by disassembling *α*-helix. *Biochemical and Biophysical Research Communications*.

[66] Liu G., Gaines J. C., Robbins K. J., Lazo N. D. (2012). Kinetic profile of amyloid formation in the presence of an aromatic inhibitor by nuclear magnetic resonance. *ACS Medicinal Chemistry Letters*.

[67] Tu L.-H., Young L. M., Wong A. G., Ashcroft A. E., Radford S. E., Raleigh D. P. (2015). Mutational analysis of the ability of resveratrol to inhibit amyloid formation by islet amyloid polypeptide: critical evaluation of the importance of aromatic–inhibitor and histidine–inhibitor interactions. *Biochemistry*.

[68] Lolicato F., Raudino A., Milardi D., la Rosa C. (2015). Resveratrol interferes with the aggregation of membrane-bound human-IAPP: a molecular dynamics study. *European Journal of Medicinal Chemistry*.

[69] Jiang P., Li W., Shea J.-E., Mu Y. (2011). Resveratrol inhibits the formation of multiple-layered *β*-sheet oligomers of the human islet amyloid polypeptide segment 22–27. *Biophysical Journal*.

[70] Wei L., Jiang P., Xu W. (2011). The molecular basis of distinct aggregation pathways of islet amyloid polypeptide. *The Journal of Biological Chemistry*.

[71] Radovan D., Opitz N., Winter R. (2009). Fluorescence microscopy studies on islet amyloid polypeptide fibrillation at heterogeneous and cellular membrane interfaces and its inhibition by resveratrol. *FEBS Letters*.

[72] Sheynis T., Friediger A., Xue W.-F. (2013). Aggregation modulators interfere with membrane interactions of *β*2-microglobulin fibrils. *Biophysical Journal*.

[73] Zhao J., Hu R., Sciacca M. F. M. (2014). Non-selective ion channel activity of polymorphic human islet amyloid polypeptide (amylin) double channels. *Physical Chemistry Chemical Physics*.

[74] Sciacca M. F. M., Kotler S. A., Brender J. R., Chen J., Lee D.-K., Ramamoorthy A. (2012). Two-step mechanism of membrane disruption by A*β* through membrane fragmentation and pore formation. *Biophysical Journal*.

[75] Zelus C., Fox A., Calciano A., Faridian B. S., Nogaj L. A., Moffet D. A. (2012). Myricetin inhibits islet amyloid polypeptide (IAPP) aggregation and rescues living mammalian cells from IAPP toxicity. *The Open Biochemistry Journal*.

[76] Noor H., Cao P., Raleigh D. P. (2012). Morin hydrate inhibits amyloid formation by islet amyloid polypeptide and disaggregates amyloid fibers. *Protein Science*.

[77] Wang H., Raleigh D. P. (2014). General amyloid inhibitors? A critical examination of the inhibition of IAPP amyloid formation by inositol stereoisomers. *PLoS ONE*.

[78] Cheng B., Gong H., Li X. (2013). Salvianolic acid B inhibits the amyloid formation of human islet amyloid polypeptideand protects pancreatic beta-cells against cytotoxicity. *Proteins: Structure, Function and Bioinformatics*.

[79] Rubio M. A., Schlamadinger D. E., White E. M., Miranker A. D. (2015). Peptide amyloid surface display. *Biochemistry*.

[80] McKoy A. F., Chen J., Schupbach T., Hecht M. H. (2012). A novel inhibitor of amyloid *β* (A*β*) peptide aggregation: from high throughput screening to efficacy in an animal model of Alzheimer disease. *The Journal of Biological Chemistry*.

[81] Chen J., Armstrong A. H., Koehler A. N., Hecht M. H. (2010). Small molecule microarrays enable the discovery of compounds that bind the Alzheimer's A*β* peptide and reduce its cytotoxicity. *Journal of the American Chemical Society*.

[82] Kim W., Kim Y., Min J., Kim D. J., Chang Y.-T., Hecht M. H. (2006). A high-throughput screen for compounds that inhibit aggregation of the Alzheimer's peptide. *ACS Chemical Biology*.

[83] Kumar S., Brown M., Nath A., Miranker A. (2014). Folded small molecule manipulation of islet amyloid polypeptide. *Chemistry & Biology*.

[84] Hebda J. A., Magzoub M., Miranker A. D. (2014). Small molecule screening in context: lipid-catalyzed amyloid formation. *Protein Science*.

[85] Kumar S., Miranker A. D. (2013). A foldamer approach to targeting membrane bound helical states of islet amyloid polypeptide. *Chemical Communications*.

[86] Brender J. R., Hartman K., Nanga R. P. R. (2010). Role of zinc in human islet amyloid polypeptide aggregation. *Journal of the American Chemical Society*.

[87] Masad A., Hayes L., Tabner B. J. (2007). Copper-mediated formation of hydrogen peroxide from the amylin peptide: a novel mechanism for degeneration of islet cells in type-2 diabetes mellitus?. *FEBS Letters*.

[88] Lee E. C., Ha E., Singh S. (2013). Copper(II)-human amylin complex protects pancreatic cells from amylin toxicity. *Physical Chemistry Chemical Physics*.

[89] Young L. M., Cao P., Raleigh D. P., Ashcroft A. E., Radford S. E. (2014). Ion mobility spectrometry-mass spectrometry defines the oligomeric intermediates in amylin amyloid formation and the mode of action of inhibitors. *Journal of the American Chemical Society*.

[90] Yesuvadian R., Krishnamoorthy J., Ramamoorthy A., Bhunia A. (2014). Potent *γ*-secretase inhibitors/modulators interact with amyloid-*β* fibrils but do not inhibit fibrillation: a high-resolution NMR study. *Biochemical and Biophysical Research Communications*.

[91] Huang R., Vivekanandan S., Brender J. R., Abe Y., Naito A., Ramamoorthy A. (2012). NMR characterization of monomeric and oligomeric conformations of human calcitonin and its interaction with EGCG. *Journal of Molecular Biology*.

[92] Hyung S.-J., DeToma A. S., Brender J. R. (2013). Insights into antiamyloidogenic properties of the green tea extract (−)-epigallocatechin-3-gallate toward metal-associated amyloid-*β* species. *Proceedings of the National Academy of Sciences of the United States of America*.

[93] del Amo J. M. L., Fink U., Dasari M. (2012). Structural properties of EGCG-induced, nontoxic Alzheimer's disease A*β* oligomers. *Journal of Molecular Biology*.

[94] Fu Z., Aucoin D., Ahmed M., Ziliox M., Van Nostrand W. E., Smith S. O. (2014). Capping of A*β*42 oligomers by small molecule inhibitors. *Biochemistry*.

